# Statecharts for Gene Network Modeling

**DOI:** 10.1371/journal.pone.0009376

**Published:** 2010-02-23

**Authors:** Yong-Jun Shin, Mehrdad Nourani

**Affiliations:** Department of Electrical Engineering, University of Texas at Dallas, Richardson, Texas, United States of America; Center for Genomic Regulation, Spain

## Abstract

State diagrams (stategraphs) are suitable for describing the behavior of dynamic systems. However, when they are used to model large and complex systems, determining the states and transitions among them can be overwhelming, due to their flat, unstratified structure. In this article, we present the use of statecharts as a novel way of modeling complex gene networks. Statecharts extend conventional state diagrams with features such as nested hierarchy, recursion, and concurrency. These features are commonly utilized in engineering for designing complex systems and can enable us to model complex gene networks in an efficient and systematic way. We modeled five key gene network motifs, simple regulation, autoregulation, feed-forward loop, single-input module, and dense overlapping regulon, using statecharts. Specifically, utilizing nested hierarchy and recursion, we were able to model a complex interlocked feed-forward loop network in a highly structured way, demonstrating the potential of our approach for modeling large and complex gene networks.

## Introduction

### Motivation

One of the main research topics of systems biology is the study of gene networks that involve the interactions between transcription factor proteins and the genes that they regulate [1]–[4]. There have been two ways of approaching this subject: bottom-up and top-down [5]. In a bottom-up approach, mathematics is used to model the dynamics, starting from detailed knowledge of the networks [6]–[9]. Live cell fluorescent reporter assay is one of the commonly used experimental techniques for the bottom-up approach [10]. On the other hand, a top-down approach aims at understanding the networks for which very limited knowledge is available [5]. Even though it is less accurate in terms of “physical quantities”, compared to the bottom-up approach, it has an advantage for dealing with large networks. For example, it can make use of expression profiling by DNA microarrays and analyze whole genome data [11]–[13].

Various mathematical and computational approaches have been developed for gene network modeling, including Boolean networks, Bayesian networks, Petri nets, ordinary differential equations, and stochastic simulation algorithms [1], [14]–[27]. These approaches can generally be grouped into two larger categories: logical and continuous models. Logical models are simple because they deal only with the logical sequence of events. On the other hand, continuous models can describe dynamics that depend on finer timing and exact molecular concentrations. Since gene expression is fundamentally stochastic, the continuous models can also include noise [19], [28]–[30]. Many dynamic systems can be approximately described using differential equations and they have been used to model the dynamics of various gene networks [9], [15], [25], [31]–[34]. Prior knowledge of system parameter values, extracted from experimental data through optimization, is required for such modeling [35].

### Logical Models

Logical models can describe gene networks qualitatively [14], [36]. Even though they are simple and easy, compared to continuous models described above, they can still allow us to obtain a basic understanding of the dynamics of complex networks. It is important to note that logical models are not generated by simple discrete approximation of the real-valued data used in continuous models. Logical models are often regarded as inferior to continuous models, based on a misunderstanding that logical models are just a simplified version of continuous models and both of them belong to the same domain. Continuous models belong to physical domain where a measurable time or quantity exists. Logical models, on the other hand, belong to a different domain, logical domain, where we are interested only in states (e.g., the presence or absence of a signal, protein, mRNA, etc.) and the sequence of state transitions (e.g., feed- forward loops described in detail later). In other words, the exact amount of physical quantities can be neglected as long as the state of entities and the sequence of state transitions are correct in logical models. Therefore, logical models are basically asynchronous, meaning that the state transitions are not confined to specific times and may occur at any time when inputs/conditions are ready/satisfied.

Boolean network, first presented by Kauffman [14], is a logical modeling approach that uses binary representation for the state of biological entities. For example, the existence of a signal can be represented as 1 (present) or 0 (absent), and the expression of a gene can also be shown as 1 (active) or 0 (inactive). Given inputs, a system may go through various transient states and eventually reach a steady state. The steady or final state and its outputs, which depend on only the input values, can be simulated and determined using combinational logic [37]. Combinational logic-based models cannot show transient or intermediate states that may have as much biological significance as the steady or final states. Therefore, there have been various approaches for capturing the transient states of gene networks, including model checking, Petri nets, Markov chain, and sequential logic [20], [23], [38]. For example, in sequential logic-based models, outputs depend on both the present state and input values [37]. In such models, even if same inputs are given, outputs can be different depending on the present state of a system, generating many different transient states. Sequential logic-based models are often represented as finite state machines and state diagrams [37]. Even though these approaches are good at showing the transient states of a dynamic system, they become less successful as the system becomes larger and more complex. For instance, in large sequential logic-based models, determining and managing the states and transitions among them can be overwhelming, due to their flat, unstratified structure. This can be resolved using statecharts as described below.

### Statecharts

Gene networks are made of a small set of recurring modules, called network motifs: (i) simple regulation (two-gene network), (ii) autoregulation, (iii) feed-forward loop, (iv) single-input module, and (v) dense overlapping regulon [4], [31]. In fact, the last four motifs are variations and/or combinations of the first motif, simple regulation. In other words, simple regulation is a fundamental unit or module of gene networks and can serve as a basic building block for constructing more complex networks. Modularity is an important property that allows scientists and engineers to model, design, or analyze complex systems in a structured and efficient way. A large system made of multiple modules can be regarded as a module for an even larger system. Also, through abstraction, the details of sub-modules (modules inside a module) can be hidden. Therefore, using modularity and abstraction, a complex, multi-level (nested) hierarchy can be implemented while maintaining simplicity.

Statechart is a sequential logic-based modeling approach that extends classical state diagram with additional features such as nested hierarchy, concurrency, and recursion, enabling us to model large gene networks in an efficient and systematic way [39]. In this paper, we will model the known network motifs described above using statecharts and describe the important features. A complex interlocked feed-forward loop network will be also modeled, demonstrating the potential of the approach for modeling large and complex gene networks.

### Understanding Simple Regulation in Continuous Domain

Understanding simple regulation, the basic building block of gene networks, in continuous (physical) domain is helpful for appreciating gene networks modeled in logical domain. A differential equation-based model of simple regulation is described in detail below.

**Figure 1:**
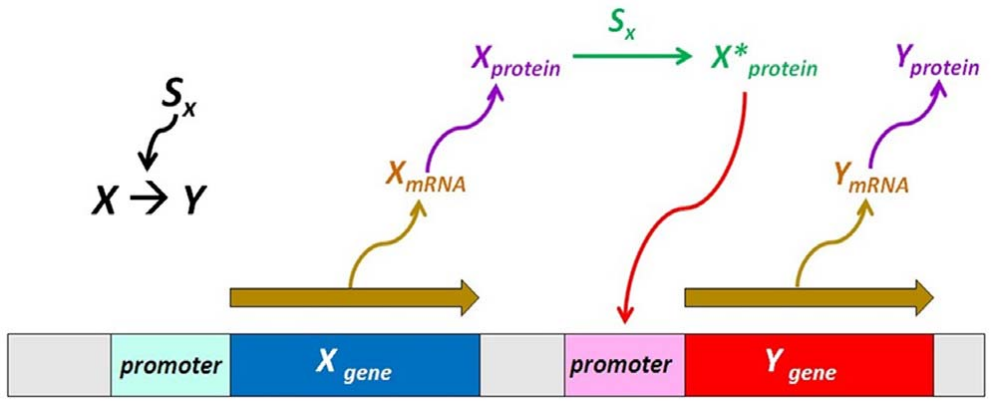
A schematic illustration of simple regulation (X→Y). First, *X_gene_* is transcribed into *X_mRNA_*, which is then translated into *X_protein_*. In the presence of signal *S_x_*, *X_protein_* transits to its active form X*_protein_ and binds the promoter of *Y_gene_,* transcribing *Y_gene_* into *Y_mRNA_*. Finally, as *Y_mRNA_* is translated, *Y_protein_* is produced.

In simple regulation, *Y_gene_* is activated by *X_gene_*, as indicated by the notation, X→Y, in Figure 1. Even though the notation is quite simple, it involves a number of steps. First, *X_gene_* is transcribed into *X_mRNA_*, which is then translated into *X_protein_*. In the presence of signal *S_x_*, *X_protein_* transits to its active form X*_protein_ and binds the promoter of *Y_gene_*, transcribing *Y_gene_* into *Y_mRNA_*. Finally, as *Y_mRNA_* is translated, *Y_protein_* is produced. Overall, the signal *S_x_* acts like a switch, controlling the rate of the *Y_protein_* production.

Depending on the concentration of X*_protein_, *Y_protein_* is formed at a rate *f(t)*, a function of time (units of concentration per unit time). The production is balanced by processes that decrease *Y_protein_*, namely degradation (protein destruction by specialized enzymes) and dilution (concentration reduction due to the increase of cell volume during growth) [31]. Degradation and dilution can be collectively denoted as *d(t)* (units of one per unit time). The change in the concentration of *Y_protein_* depends on both *f(t)* and *d(t)*. Using a differential equation, its dynamics can be described as:
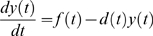
where *y(t)* stands for the concentration of *Y_protein_*.

As stated earlier, *X_protein_* must be converted to X*_protein_ by the signal *S_x_* in order to initiate the *Y_protein_* production. The concentration of X*_protein_ can be expressed as a function of *S_x_*, which is acting as an activating switch. The elements of biological systems that have switch-like relationships with one another can be described using the Hill function [40]. Thus, the relationship between X*_protein_ and *S_x_* can be expressed as:
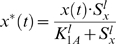
where *x*(*t*) stands for the concentration of *X_protein_*. It is the maximal level of X*_protein_ or x*(t) (in units of concentration) that is reached when *S_x_*≫*K_1A_*. *K_1A_* is the concentration of *S_x_*, at which half-maximal concentration of x*(t) is reached. The Hill coefficient *l* changes the steepness of the function. When *S_x_* acts as a repressor, the Hill function can be expressed as:
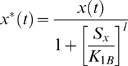
where *K_1B_* is the concentration of *S_x_*, at which half-maximal repression of the x*(t) production is reached.

The relationship between *f(t)* and X*_protein_ (*S_x_*) or x*(t) has also been experimentally demonstrated as [33]:
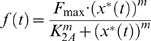



*F_max_* is the maximal level of the *Y_protein_* production (in units of concentration per unit time) that is reached when x*(t)≫*K_2A_*. *K_2A_* is the concentration of x*(t) at which half-maximal production of *Y_protein_* is reached. Again, *m* is the Hill coefficient. Similarly, when x*(t) acts as a repressor, the Hill function can be shown as:
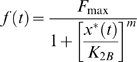
where *K_2B_* is the concentration of x*(t) at which half-maximal repression of the *Y_protein_* production is reached.

## Results and Discussion

### Simple Regulation

**Figure 2:**
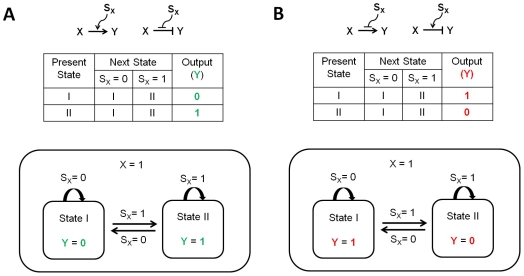
Simple regulation. (A) Coherent simple regulation. Both the signal *S_X_* and transcription factor *X* act either as activators or repressors. (B) Incoherent simple regulation. One of them acts as an activator and the other acts as a repressor.

Figure 2 shows the state tables and statecharts of various simple regulations. The existence of the signal *S_x_* is denoted by 1 (present) and 0 (absent). The expression state of gene *X* and *Y* is also represented as 1 (active) and 0 (inactive). It is assumed that gene *X* is always expressed (*X* = 1). The signal *S_x_* and transcription factor *X* can act both as activators or repressors. Therefore, for simple regulation, there are four possible combinations regarding the actions of the signal and transcription factor. When they act in the same way (both of them are either activators or repressors), it is called a coherent simple regulation (Fig. 2A). On the other hand, if the signal and transcription factor behave in opposite ways (one is an activator while the other one is a repressor), it is an incoherent simple regulation (Fig. 2B). Note that both State I and II are within a larger state where *X* = 1, forming a nested hierarchical structure. The large state (*X* = 1) is a superstate, and state I and II are substates. The current state of the signal *S_x_* determines which substate is an initial (or starting) substate. For example, in Figure 2A, if *S_x_* = 1 then the right substate is considered first during execution.

### Autoregulation

**Figure 3:**
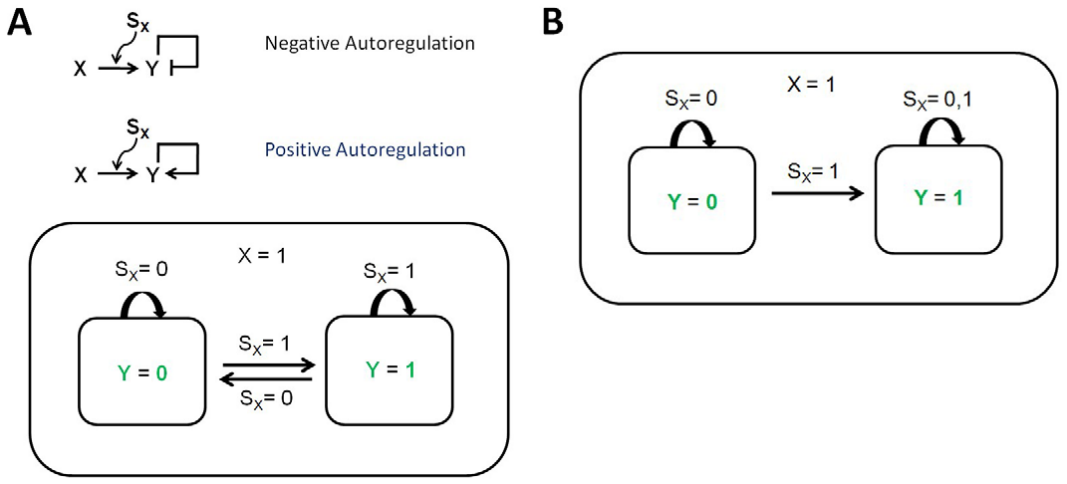
Autoregulations. (A) Both negative and positive autoregulations are identical to simple regulation in logical domain. (B) Positive autoregulation may lock the network into a state where gene *Y* is constantly expressed.

Negative autoregulation occurs when a transcription factor represses the transcription of its own gene (negative feedback). It is known that negative autoregulation speeds up the response time of gene expression and reduce the cell-cell variation in protein levels [31]. Positive autoregulation occurs when a transcription factor enhances its own protein production rate. In contrast to negative autoregulation, the response time is slowed and the cell-cell variation is increased [31]. Both negative and positive autoregulations are identical to simple regulation in logical domain because only the state of entities and the sequence of state transitions are considered, neglecting all the physical details such as the response time and cell-cell variation (Fig. 3A). It has been reported that when the rate of positive autoregulation is very strong compared to the degradation/dilution rate, the network can be locked in one state [31], [41], [42]. In other words, the expression state of gene *Y* may become irreversible once it is activated, even after the signal is no longer present, as shown in Figure 3B.

### Feed-Forward Loop (FFL)

Feed-forward loop (FFL) is one of the most studied motif classes [4], [32], [43], [44]. Among many types within the FFL class, coherent type-1 (C1-FFL) and incoherent type-1 (Ic1-FFL) are the ones commonly found in biological systems [31].

**Figure 4:**
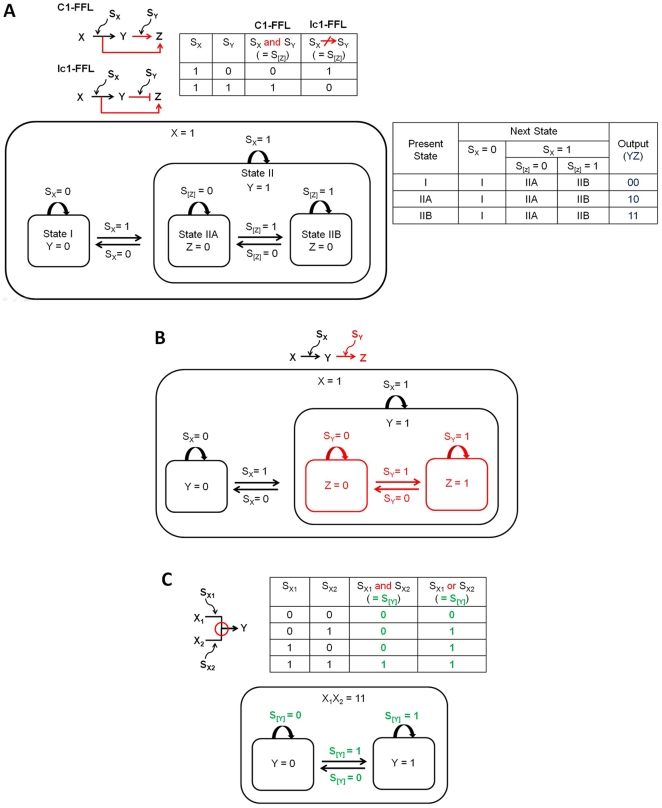
Feed-forward loops. (A) The statechart and state table for C1-FFL and Ic1-FFL. The truth table for the signals is also shown. (B) Cascaded simple regulations (C) Junction rule based on combinational logic. S_[Y]_ signifies that multiple signals are acting on gene *Y* and the net effect is determined by their combinational logic-based rule.

Figure 4A shows the statechart and state table of C1-FFL and Ic1-FFL. It is known that C1-FFL causes a delay and Ic1-FFL generates a pulse in the expression of gene *Z* [31]. The FFLs are equivalent to two cascaded simple regulations (Fig. 4B) with a junction at *Z*, upon which both *X* and *Y* are acting. As shown in both Figure 4A and 4B, cascaded simple regulations do not lose the basic structure of simple regulation (Fig. 2) through recurring hierarchical organization (recursion), decreasing the complexity of the model. Figure 4C shows a combinational logic-based rule that determines the overall effect of multiple signals at the junction. Only “and” and “or” are shown in the figure, however, theoretically all other known Boolean logic gates, such as “xor” and “nand”, can also be applied [37]. S_[Y]_ signifies that multiple signals are acting on gene *Y* and the net effect is determined by their combinational logic-based rule. In Figure 4A, it is shown that C1-FFL involves “and” gate and Ic1-FFL is dependent on “doesn't imply” gate. Note that once these junction gates are defined, the statechart is identical for both types of FFLs.

**Figure 5:**
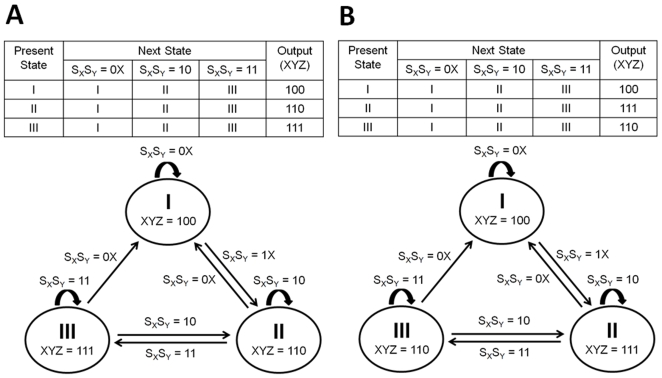
State diagrams of FFLs. (A) The state table and state diagram of C1-FFL. The symbol X (denoting “don't care”) for the signal indicates that it can be either 1 or 0. (B) The state table and state diagram of Ic1-FFL.

Figure 5 shows the state tables and state diagrams of FFLs. Note that the characteristic features of statecharts, such as nested hierarchy and recursion, cannot be seen in the state diagrams. We will show later (using an interlocked FFL gene network as an example) that the lack of those features increases the complexity of determining the states and drawing the transitions among them, as the network becomes larger.

### Single-Input Module (Parallel Simple Regulations)

**Figure 6:**
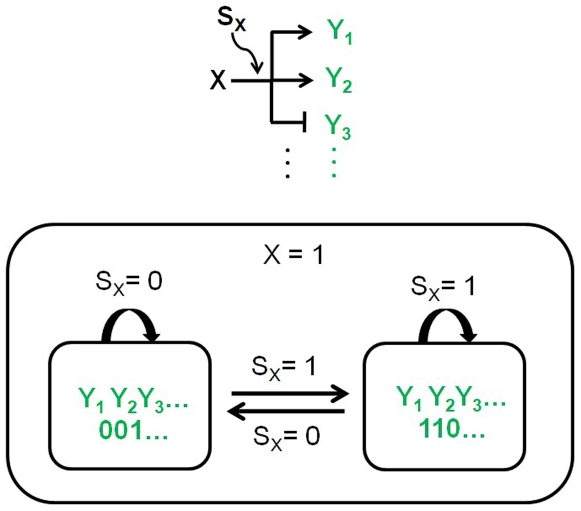
Single-input module. Multiples genes (*Y_1_*, *Y_2_*, *Y_3_*, …) are controlled by a single gene *X*.

In a single-input module, multiples genes (*Y_1_*, *Y_2_*, *Y_3_*, …) are controlled by a single gene *X* (Fig. 6). It can be considered as parallel simple regulations, in contrast to cascaded simple regulations shown in Figure 4B. The structure of the statechart is basically identical to that of simple regulation (Fig. 2), maintaining simplicity. Parallel and cascaded simple regulations are two major interconnection topologies or configurations that can make gene networks complex and diverse.

### Dense Overlapping Regulon

**Figure 7:**
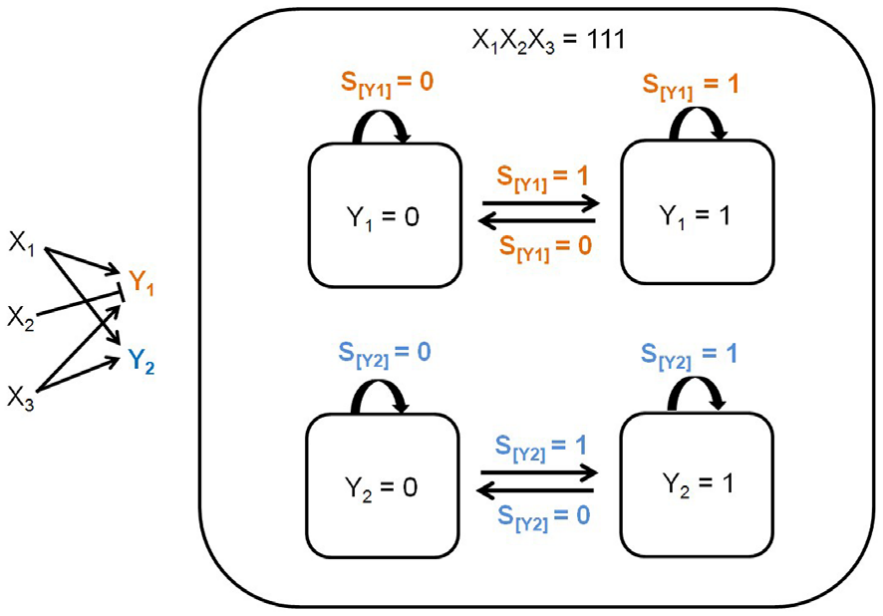
Dense overlapping regulon. Two simple regulations with combinational logic-based junctions (*S_[Y1]_* and *S_[Y2]_*) are within the same state (*X_1_X_2_X*_3_ = 111). While the superstate (*X_1_X_2_X*_3_) is active, two statecharts (each involving *Y_1_* or *Y_2_*) are executed in parallel. It is not shown in the figure, but the signals that determine *S_[Y1]_* and *S_[Y2]_*, based on combinational logic, are *S_x1_*, *S_x2_*, and *S_x3_*.

Figure 7 shows an example of dense overlapping regulon. Two simple regulation-like diagrams with combinational logic-based junctions (*S_[Y1]_* and *S_[Y2]_*) are placed within a superstate (*X_1_X_2_X*_3_ = 111). This example shows one of the important features of statecharts, concurrency. While the superstate (*X_1_X_2_X*_3_) is active, two statecharts (each involving *Y_1_* or *Y_2_*) are executed in parallel. It is not shown in the figure, but the signals that determine *S_[Y1]_* and *S_[Y2]_*, based on combinational logic, are *S_x1_*, *S_x2_*, and *S_x3_*. Note that the number of simple regulation-like diagrams depends on the number of *Y*-level genes (*Y_1_*, *Y_2_*, …) and not *X*-level genes.

### Interlocked FFL Network

**Figure 8:**
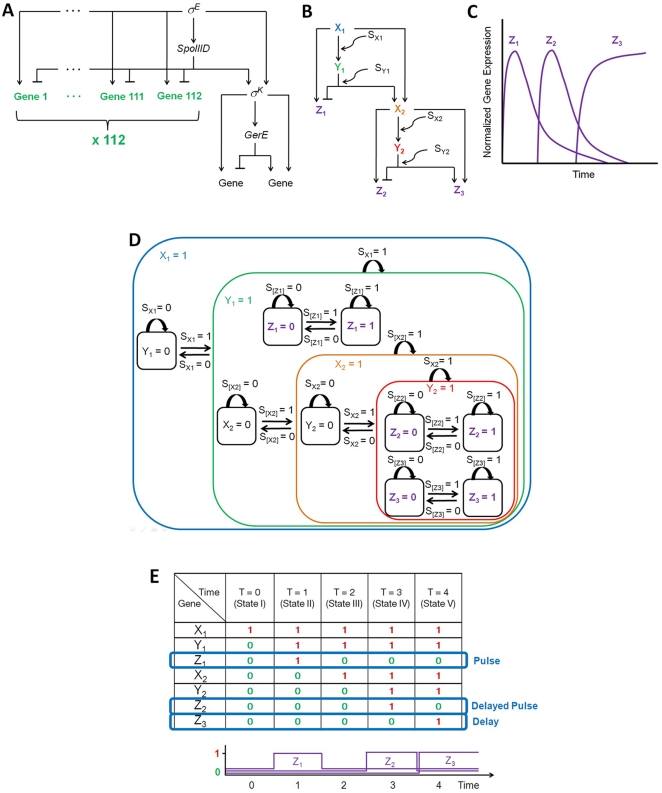
Interlocked FFL network. (A) In *Bacillus subtilis.* (B) A simplified schematic illustration. (C) Time-dependent gene expression of *Z*_1_, *Z*_2_, and *Z*_3_. (D) Statechart based on (B). [Z1] and [Z2] follow “does not imply” gate logic, while [X2] and [Z3] follow “and” gate logic. (E) Time-dependent gene expression given all the signals (S_X1_, S_Y1_, S_X2_, and S_Y2_) turned on simultaneously. *Z_1_* and *Z_2_* pulses and delayed *Z_2_* and *Z_3_* expression can be observed as shown in (C). These pulses and delays are observable because the statecharts enable us to track the transient states (states I through VI) during execution.

It is known that FFLs can be combined into more complex and larger transcription networks [4]. One example is found in the bacterium *Bacillus subtilis* where the network controls differentiation [45]. Figure 8A shows the network from the literature. The network is made up of many repeating C1-FFLs and Ic1-FFLs. However, only 112 FFLs, which are clearly described as repeating Ic1-FFLs in the literature, are included in the figure. Figure 8B shows a simplified schematic illustration of the network. Since 112 genes behave in the same way (incoherent type-1 feed-forward loop), we denote them simply as *Z*_1_, simplifying the representation. The behavior of each gene can be understood using the concept of single-input module (parallel simple regulations) described earlier. Figure 8C shows time-dependent gene expression pattern of *Z_1_*, *Z_2_*, and *Z_3_* [4]. Two Ic1-FFLs generate pulses in the expression of *Z_1_* and *Z_2_*, and two C1-FFLs cause delays in the *Z_2_* and *Z_3_* expression.

Figure 8D shows the statechart of the network. The total number of genes involved in the network is 118 (Fig. 8A). It is striking that the total number of all the superstates and substates is greatly reduced, even though the number of all the possible gene expression combinations is 2^117^ (not 2^118^ because it is assumed that the expression state of the first gene *X_1_* is on (*X_1_* = 1), which is approximately 1.66×10^35^. Note that parallel simple regulations discussed in Single-Input Module section makes this reduction possible. Furthermore, the important features (nested hierarchy, recursion, and concurrency) of statecharts discussed in previous sections are well demonstrated in this example.

When all the signals (S_X1_, S_Y1_, S_X2_, and S_Y2_) are turned on simultaneously, time-dependent gene expression can be shown as in Figure 8E. *Z_1_* and *Z_2_* pulses and delayed *Z_2_* and *Z_3_* expression are observed as expected in Figure 8C. These pulses and delays are observable because the statecharts enable us to track the transient states (states I through VI) during execution.

**Figure 9:**
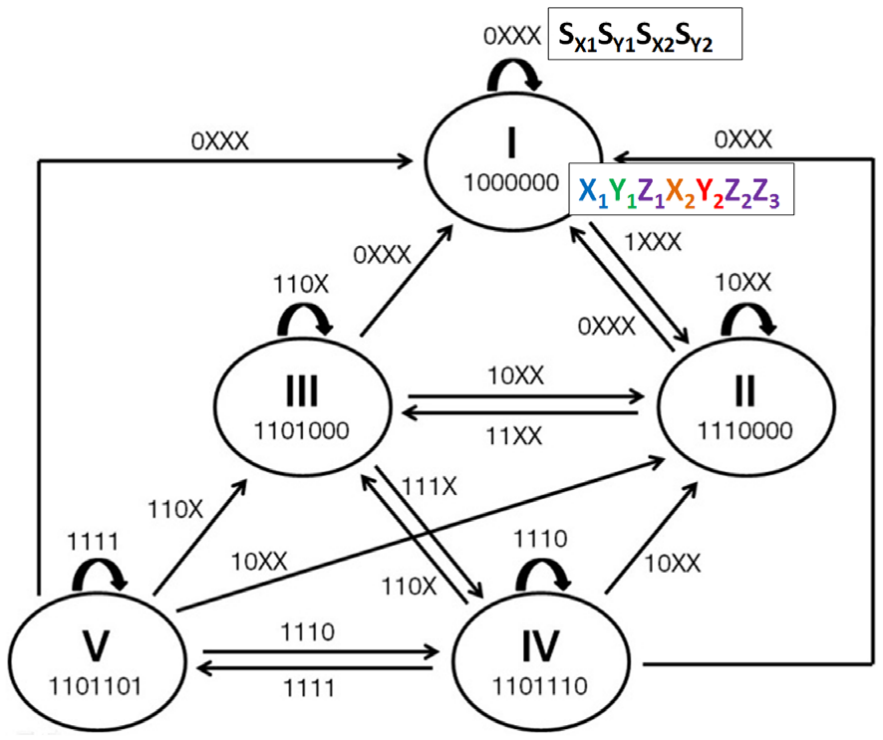
State diagram of the interlocked FFL network. Determining the states and drawing the transitions between them are more complicated compared to the statechart-based approach.

Figure 9 shows the state diagram of the same gene network. When we try to draw the state diagram, two major difficulties, compared to the statechart-based approach, become evident. First, in order to determine the states shown in the figure, we have to know the expression state of all genes for each state. This can be overwhelming if the number of genes is large. In contrast, knowing the expression state of a single gene per state is required in the statechart method (Fig. 8D). Secondly, in the state diagram, we need to consider every transition from one state to another, depending on every possible combination of the signals. Figure 9 shows that it makes the transition map very complicated. This chaotic transition problem is not seen in statecharts (Fig. 8D). In summary, determining the states and figuring out transitions between them can become daunting problem as the network size increases, and they can be handled in a more structured and efficient way using statecharts [39].

### Further Discussions

Feedback loop is not described as one of the motifs in the literature [31], and its statechart representation is not demonstrated in our paper. However, considering its importance, we intend to incorporate it into our modeling scheme in the future.

Figure 8E resembles time-series DNA microarray data, and it suggests that our approach may be useful for extracting network information from the data. In order to apply such approach, the first thing that needs to be done is extracting binary information (on and off state of each gene at different times) from experimental microarray data. However, even this is quite challenging currently because of the stochastic nature of the data and other reasons [46].

### Conclusion

The dynamics of gene networks depend on both the present signal values and the past behavior of the system, and sequential logic-based state diagrams are appropriate for representing such dynamics. However, when they are used to model large, complex systems, determining the states and managing transitions between them can become chaotic and unrealistic. In this article, we demonstrated how statecharts, which extend state diagrams with features including nested hierarchy, recursion, and concurrency, enable us to model large gene networks in a highly structured and efficient way. We modeled five known gene network motifs, simple regulation, autoregulation, feed-forward loop, single-input module, and dense overlapping regulon, using the statechart method. Utilizing the important features of statecharts, we were also able to model a complex interlocked feed-forward loop network, demonstrating the potential of our approach for modeling large and gene networks.

## Methods

The statechart method used in this article is described in detail in Results and Discussion Section. It was first invented by David Harel in 1980s, and a detailed introduction to the subject can also be found in one of his papers [39].
